# Supplementary material for: Resolution of SLC6A1 variable expressivity in a multi-generational family using deep clinical phenotyping and Drosophila models
Source: medRxiv. 2024 Sep 28:2024.09.27.24314092. Preprint. [Version 1] doi: 10.1101/2024.09.27.24314092 (PMC11469343; doi:10.1101/2024.09.27.24314092)
Supplement: Supplement 1 — Figure S1 Phenotypic depth. The number of HPO terms per individual is plotted. On average, there are 12 HPO terms per individual noted for further analysis. [file media-1.pdf]

Supplemental Figures

Figure S1

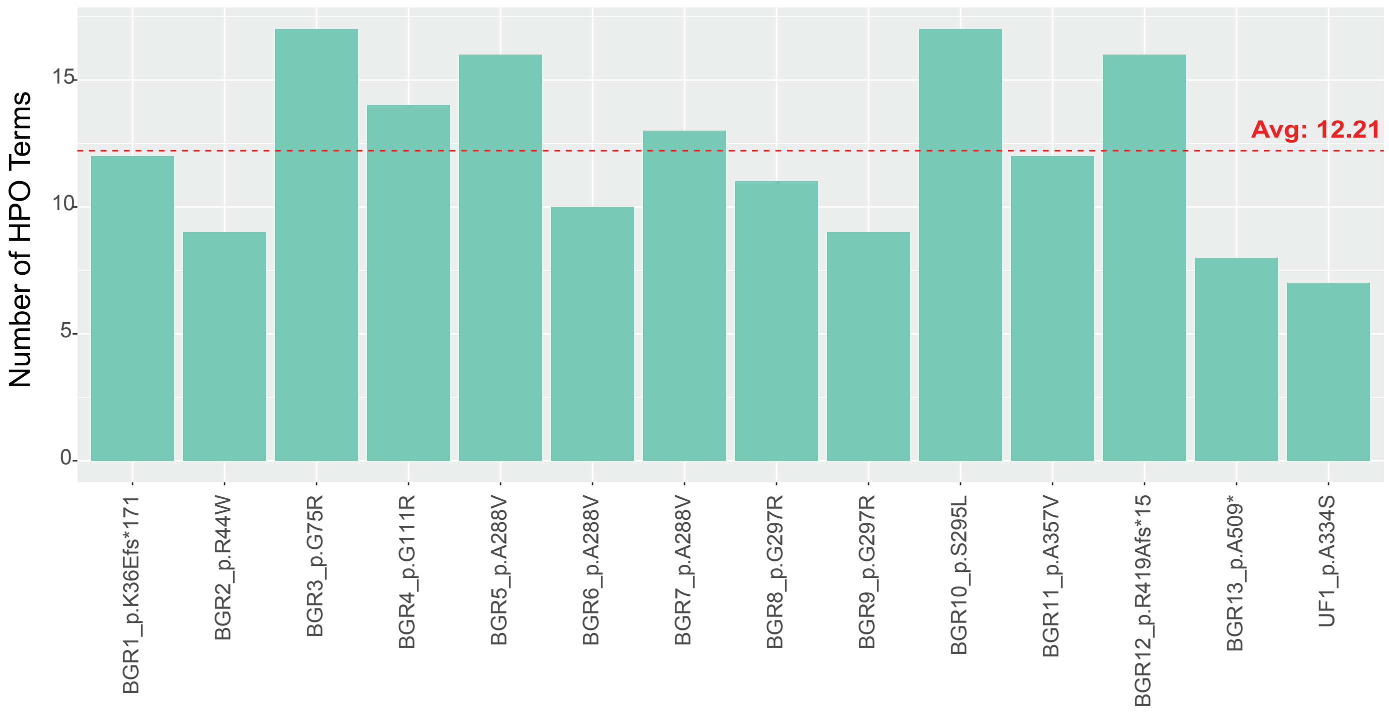

Figure S1: Phenotypic depth. The number of HPO terms per individual is plotted. On average, there are 12 HPO terms per individual noted for further analysis.
