# Supplementary material for: Resolution of SLC6A1 variable expressivity in a multi-generational family using deep clinical phenotyping and Drosophila models
Source: medRxiv. 2024 Sep 28:2024.09.27.24314092. Preprint. [Version 1] doi: 10.1101/2024.09.27.24314092 (PMC11469343; doi:10.1101/2024.09.27.24314092)
Supplement: Supplement 2 — Figure S2 RNAP domain scores. Seven RNAP domain scores (%) are plotted along with percentage of answered questions for each individual per domain. A greater domain score indicates a worse phenotype noted in the individual. [file media-2.pdf]

Supplemental Figures

Figure S2

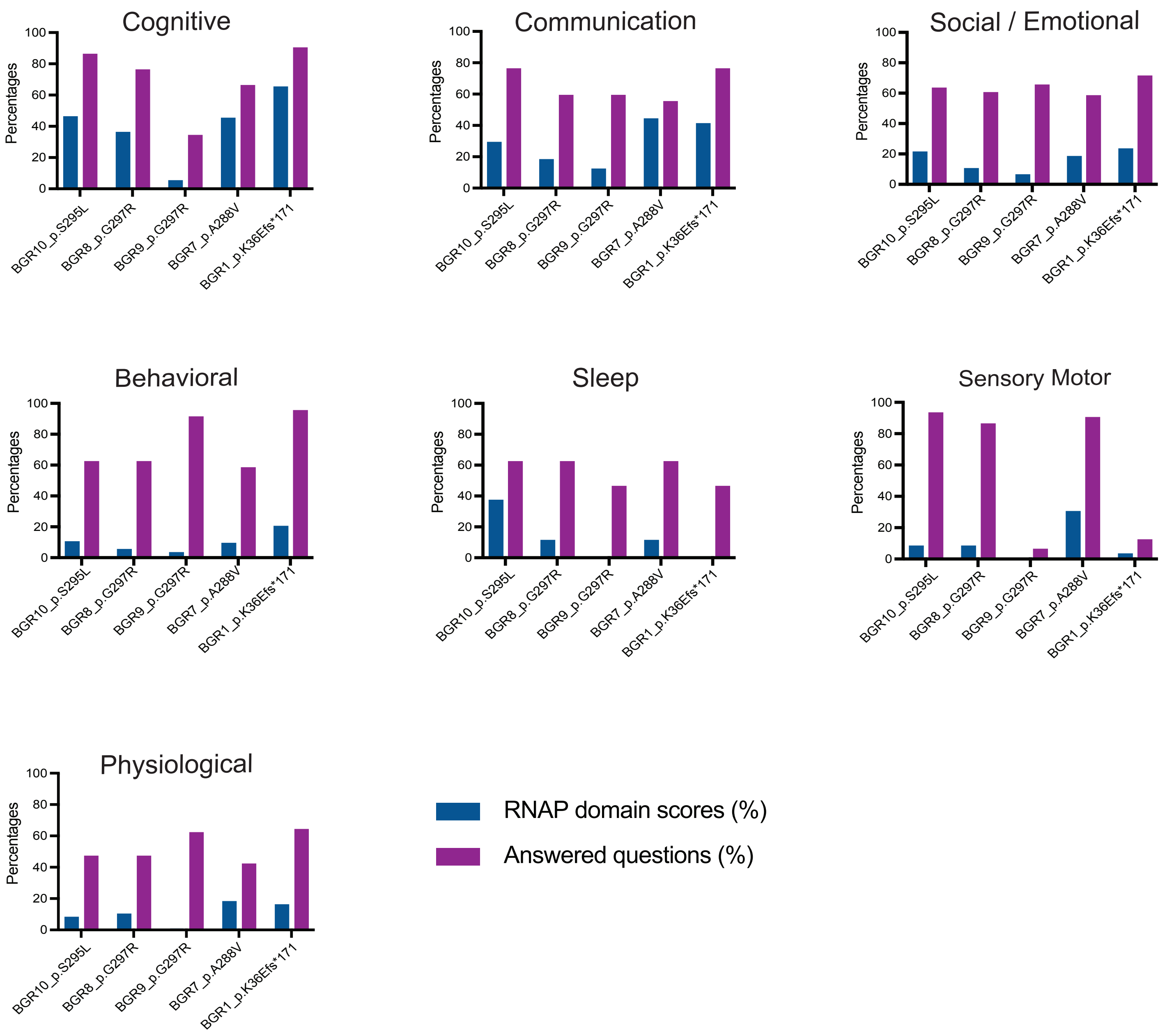

Figure S2: RNAP domain scores. Seven RNAP domain scores (%) are plotted along with percentage of answered questions for each individual per domain. Greater the percentage of domain score wrose the phenotype noted in the individual.
