# Supplementary material for: Resolution of SLC6A1 variable expressivity in a multi-generational family using deep clinical phenotyping and Drosophila models
Source: medRxiv. 2024 Sep 28:2024.09.27.24314092. Preprint. [Version 1] doi: 10.1101/2024.09.27.24314092 (PMC11469343; doi:10.1101/2024.09.27.24314092)
Supplement: Supplement 3 — Figure S3 Annotation grid of BGR8_p.G297R. This is a case of dual diagnosis with variants in both SLC6A1 and PTPN11, both explain the totality of the phenotypes noted in the individual. [file media-3.pdf]

Supplemental Figures

Figure S3

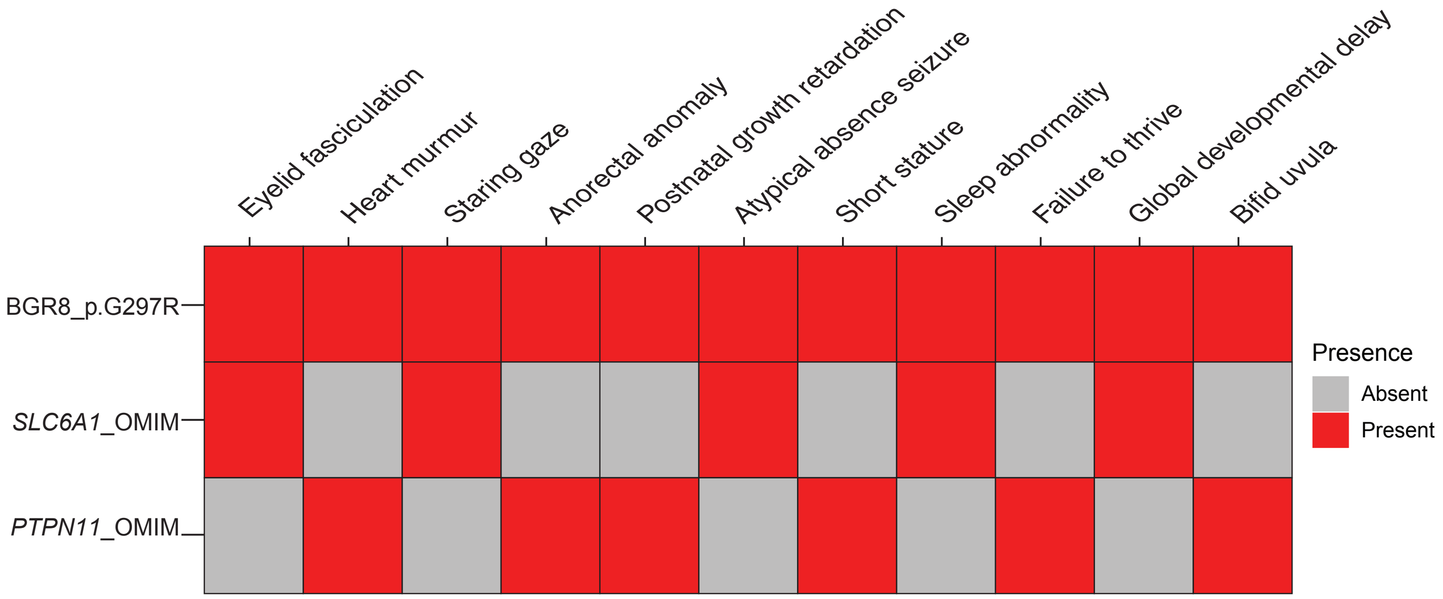

Figure S3: Annotation grid of BGR_Family08_p.G297R. This is a case of dual diagnosis with variants in both *SLC6A1* and *PTPN11*, both explain the totality of the phenotypes noted in the individual.
