# Supplementary figures and images for: Resolution of SLC6A1 variable expressivity in a multi-generational family using deep clinical phenotyping and Drosophila models
Source: medRxiv. 2024 Sep 28:2024.09.27.24314092. Preprint. [Version 1] doi: 10.1101/2024.09.27.24314092 (PMC11469343; doi:10.1101/2024.09.27.24314092)

## Seizure recovery

A

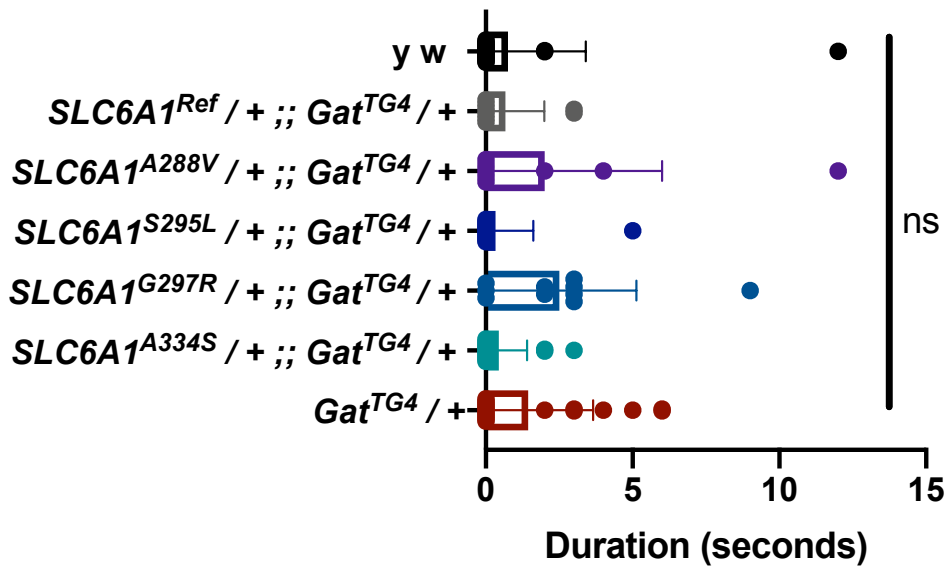

## Seizure recovery

B

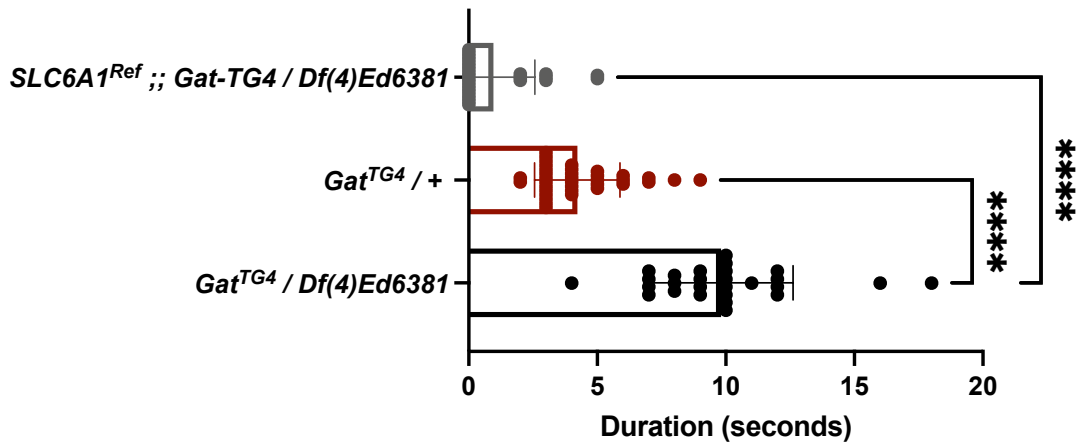

## Longevity

C

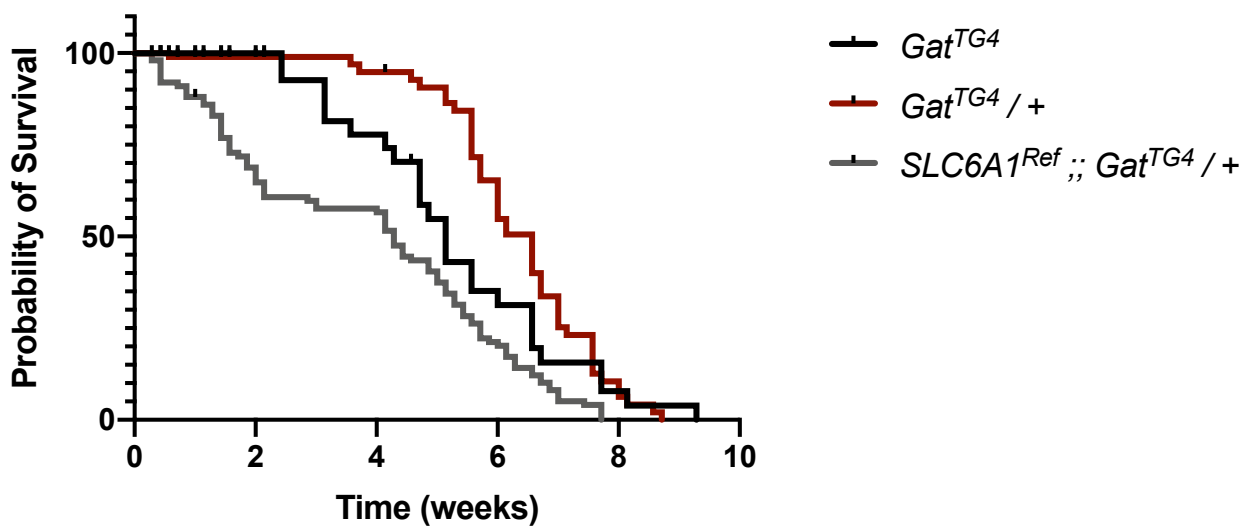

Supplement: Supplement 4 — Figure S4 Human SLC6A1 suppresses bang sensitivity in flies. (A). Expression of SLC6A1Ref, SLC6A1A288V, SLC6A1S295L, SLC6A1G297R, or SLC6A1A334S with GatTG4 does not result in seizure sensitivity. (B) Homozygous loss of Gat (GatTG4 / Df(4)Ed6381) induces bang sensitivity that is rescued by co-expression of SLC6A1Ref. (C) Loss of Gat results in reduced longevity, and expression of SLC6A1Ref with GatTG4 induces a more severe reduced longevity phenotype. [file media-4.pdf]

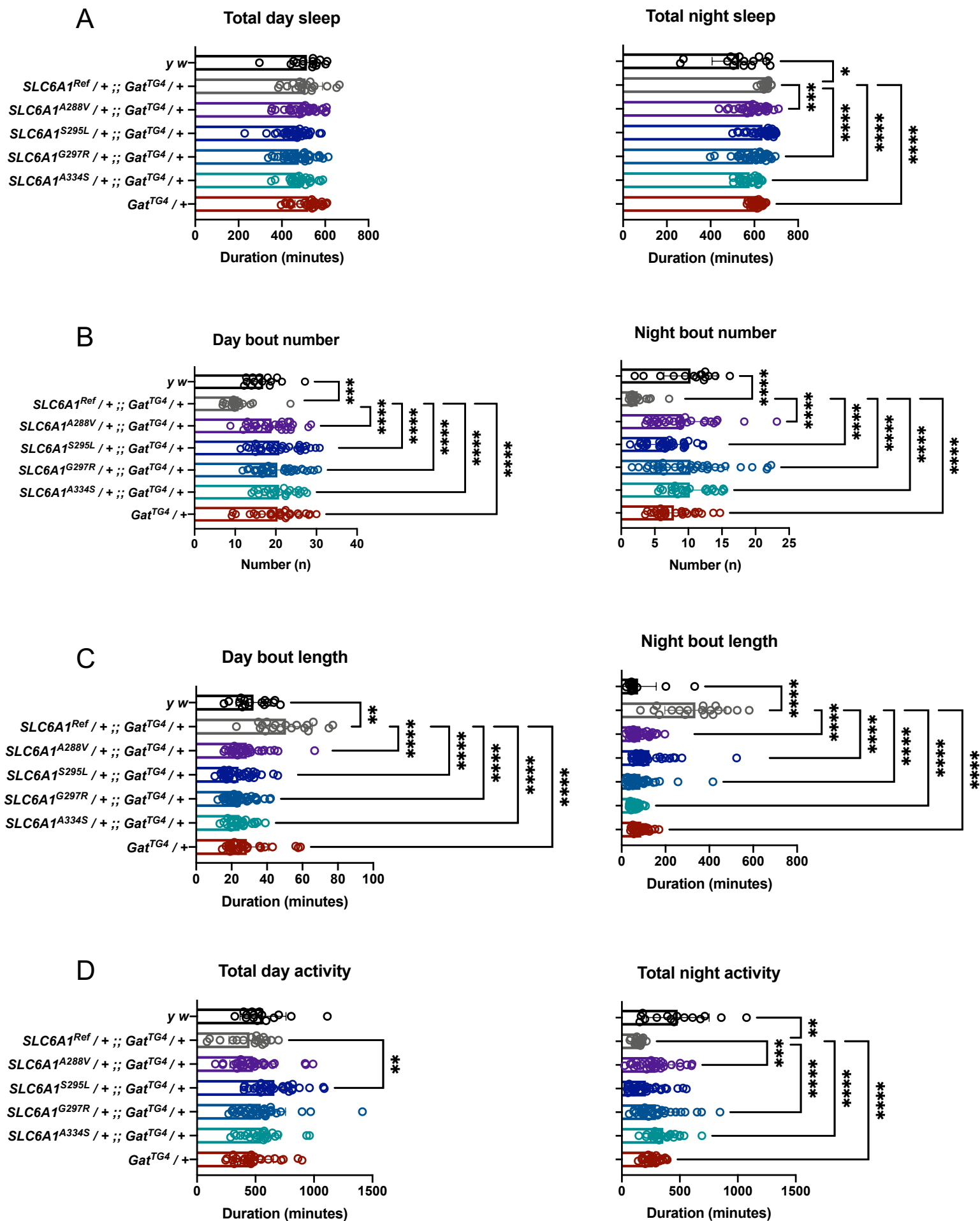

Supplement: Supplement 5 — Figure S5 Day and night sleep are more fragmented when SLC6A1A334S is expressed. (A) Total day sleep is not affected but total night sleep is decreased for SLC6A1A288V (P=0.011), SLC6A1G297R (P<0.0001), SLC6A1A334S (P<0.0001). (B) Sleep bout number is increased during the day for SLC6A1A288V (P<0.0001), SLC6A1S295L (P<0.0001), SLC6A1G297R (P<0.0001), SLC6A1A334S (P<0.0001) and night SLC6A1A288V (P<0.0001), SLC6A1S295L (P<0.0001), SLC6A1G297R (P<0.0001), and SLC6A1A334S (P<0.0001). (C) Sleep bout length is decreased during the day for SLC6A1A288V (P<0.0001), SLC6A1S295L (P<0.0001), SLC6A1G297R (P<0.0001), and SLC6A1A334S (P<0.0001) and night SLC6A1A288V (P<0.0001), SLC6A1S295L (P<0.0001), SLC6A1G297R (P<0.0001), and SLC6A1A334S (P<0.0001). (D) Total activity is increased during the day for SLC6A1S295L (P=0.0014) and increased during the night for SLC6A1A288V (P=0.0001), SLC6A1G297R (P<0.0001), SLC6A1A334S (P<0.0001). Welch’s ANOVA with Dunnett’s T3 multiple comparisons test. [file media-5.pdf]

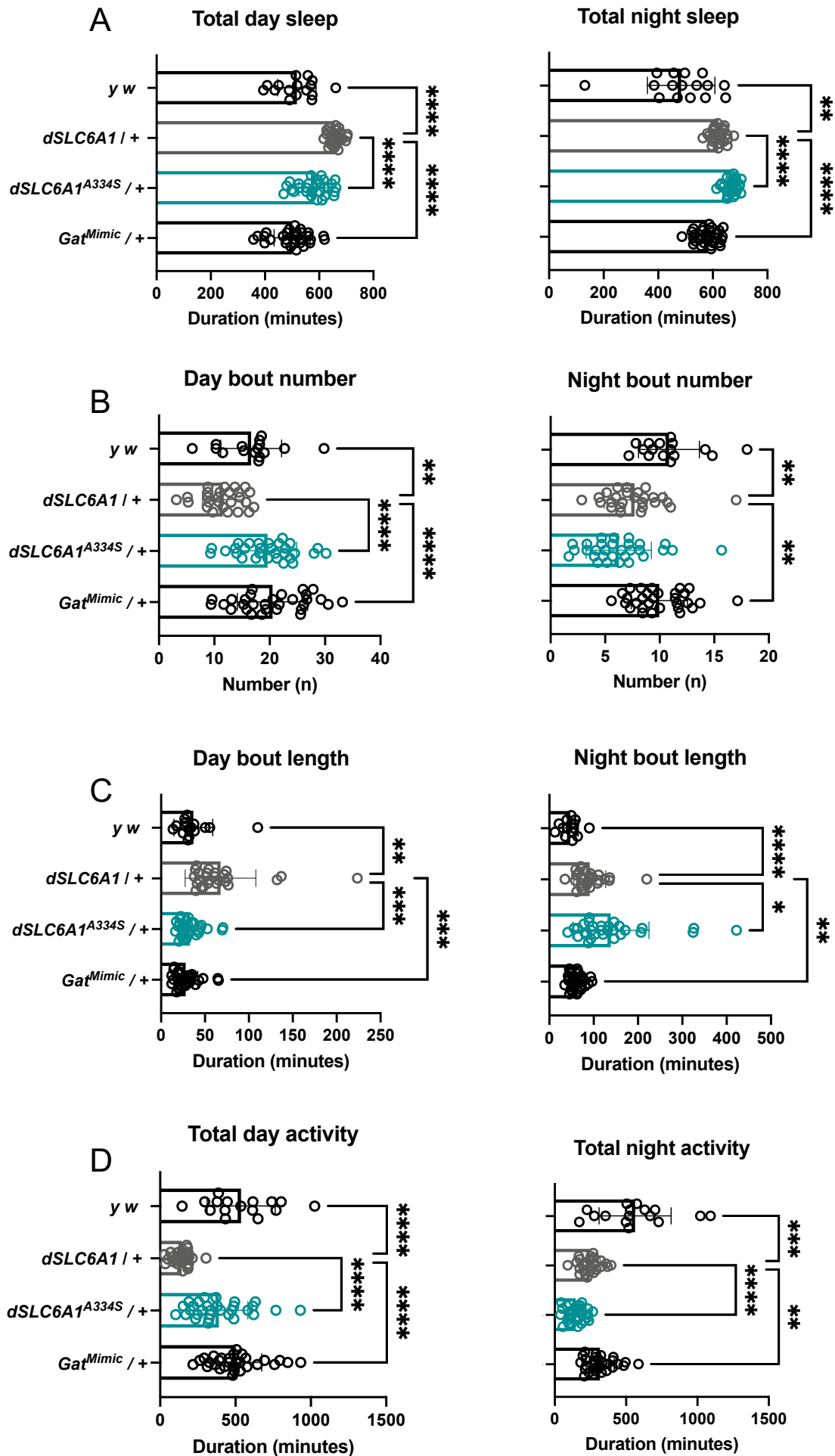

Supplement: Supplement 6 — Figure S6 Expression of the UDN variant promotes night sleep consolidation. (A) There is a decrease in dSLC6A1A334S total day sleep (P<0.0001) and an increase in total night sleep (P<0.0001) compared to dSLC6A1. (B) There is an increase in dSLC6A1A334S day sleep bout number (P<0.0001) and no significant change in night bout number. (C) There is a decrease in dSLC6A1A334S day sleep bout length (P=0.0006) and an increase in night bout length (P=0.0254). (D) There is an increase in dSLC6A1A334S total day activity (P<0.0001) and a decrease in total night activity dSLC6A1A334S (P<0.001). Welch’s ANOVA with Dunnett’s T3 multiple comparisons test. [file media-6.pdf]
